# Pasteurized *Akkermansia muciniphila* HB05 (HB05P) Improves Muscle Strength and Function: A 12-Week, Randomized, Double-Blind, Placebo-Controlled Clinical Trial

**DOI:** 10.3390/nu16234037

**Published:** 2024-11-26

**Authors:** Chang-Ho Kang, Eun-Soo Jung, Su-Jin Jung, Yeon-Hee Han, Soo-Wan Chae, Do Yeun Jeong, Byoung-Chan Kim, Seung-Ok Lee, Sun-Jung Yoon

**Affiliations:** 1 HealthBiome, Co., Ltd., 125 Gwahak-ro, Yuseong-gu, Daejeon 34141, Republic of Korea; changho-kang@healthbiome.co.kr (C.-H.K.); dyjeong@healthbiome.co.kr (D.Y.J.); bckim@healthbiome.co.kr (B.-C.K.); 2 Clinical Trial Center for Functional Foods, Jeonbuk National University Hospital, Jeonju 54907, Republic of Korea; esjung@jbctc.org (E.-S.J.); sjjeong@jbctc.org (S.-J.J.); swchae@jbctc.org (S.-W.C.); 3 Biomedical Research Institute, Jeonbuk National University Hospital, Jeonju 54907, Republic of Korea; yani0878@jbnu.ac.kr; 4 Department of Nuclear Medicine, Jeonbuk National University Medical School, Jeonju 54907, Republic of Korea; 5 Department of Nuclear Medicine, Jeonbuk National University Hospital, Jeonju 54896, Republic of Korea; 6 Department of Gastroenterology and Hepatology, Jeonbuk National University Medical School, Jeonju 54896, Republic of Korea; 7 Department of Orthopedic Surgery, Jeonbuk National University Medical School, 567 Baekje-daero, Jeonju 54896, Republic of Korea

**Keywords:** *Akkermansia muciniphila*, sarcopenia, muscle atrophy, muscle strength, follistatin

## Abstract

Background/Objectives: Sarcopenia, a condition marked by muscle wasting due to aging or inactivity, severely affects older populations. We previously showed that pasteurized *Akkermansia muciniphila* HB05 (HB05P), sourced from the breast milk of healthy Korean women, could mitigate muscle wasting in a dexamethasone-induced rat model. Here, we explored whether the oral administration of HB05P can enhance muscle strength and functionality in elderly individuals. Our objective was to determine if HB05P supplementation could benefit muscle performance in aging adults. Methods: We conducted a 12-week, double-blind, placebo-controlled clinical trial involving 100 individuals aged 60 and above, randomly assigned to receive either HB05P (1.0 × 10^10^ cells/day) or a placebo. Results: The HB05P group showed significant improvements in peak torque and peak torque per body weight of the left leg extensor muscles compared to the placebo group (*p* = 0.0103 and *p* = 0.0052). Furthermore, HB05P notably elevated follistatin levels, which counteract myostatin, relative to the placebo group (*p* = 0.0063). No notable safety concerns arose between the groups. Conclusions: HB05P is a promising postbiotic derived from *Akkermansia muciniphila* that may enhance muscle strength and be used as a safe postbiotic ingredient of *Akkermansia muciniphila* to improve muscle health.

## 1. Introduction

Sarcopenia, a condition linked to aging, is marked by a continuous reduction in muscle mass and strength. This decline significantly impacts the quality of life in elderly individuals and heightens the likelihood of chronic diseases [1]. The underlying causes of muscle degeneration are multifaceted and include hormonal imbalances, persistent inflammation, and insulin resistance [2]. Reduced muscle mass contributes to decreased physical activity, creating a self-reinforcing cycle of further muscle deterioration [3]. Research indicates that, starting at age 50, muscle strength and mass decrease by 1–2% annually, with the loss rate accelerating to around 3% per year after age 60 [4]. Currently, no pharmaceutical treatments for sarcopenia have been approved, leaving dietary protein, nutritional supplements, and resistance training as the primary approaches to maintaining muscle health [5,6]. Advances in microbiome research have led to investigations into the use of probiotic strains, such as *Lactobacillus plantarum* HY7715, *Lactobacillus plantarum* TWK10, heat-inactivated *Bifidobacterium breve* B-3, *Faecalibacterium prausnitzii*, and *Lactobacillus gasseri* BNR17, as potential therapeutic options [7,8,9,10].

Muscle atrophy, a condition driven by an imbalance between protein synthesis and degradation, can be precipitated by factors such as aging, disease, or prolonged inactivity [11]. Key pathways involved in muscle protein degradation include autophagy and the ubiquitin–proteasome system [11], with muscle-specific E3 ubiquitin ligases, namely MuRF-1 and Atrogin-1, playing essential roles under catabolic states like cachexia [12,13,14]. The regulation of these ligases is influenced by myostatin, a negative regulator of muscle hypertrophy, whereas follistatin, encoded by the FST gene, is an antagonist to myostatin, facilitating muscle growth [15,16,17]. Experimental models, such as myostatin-deficient mice [18,19], have demonstrated that follistatin overexpression leads to significant muscle hypertrophy, and the systemic administration of follistatin in animal studies has shown promising effects in mitigating muscle wasting [18,20]. 

*Akkermansia muciniphila*, a prominent commensal bacterium in the human gut, has been widely acknowledged for its role in enhancing metabolic health, maintaining gut barrier integrity, and modulating inflammation [21,22,23,24]. It produces short-chain fatty acids (SCFAs) such as acetate, propionate, and butyrate and contributes to mucin degradation, which supports metabolic and immune homeostasis [25,26,27]. Both live and pasteurized *A. muciniphila* exhibit notable anti-inflammatory and anti-fibrotic effects, with pasteurization providing advantages in stability and safety [28,29,30,31]. Pasteurized *A. muciniphila* HB05 (HB05P) can influence the expression of Atrogin-1 and follistatin in muscle cells under catabolic stress and enhance grip strength in a rat model of muscle atrophy [32]. Comprehensive preclinical safety assessments conducted in compliance with Good Laboratory Practice (GLP) standards have confirmed the safety of HB05P. 

Based on our earlier finding that HB05P can mitigate muscle wasting in a dexamethasone-induced rat mode, here we explored whether the oral administration of HB05P can enhance muscle strength and functionality in elderly individuals. Our objective was to determine if HB05P supplementation could benefit muscle performance in aging adults. In this study, HB05P was orally administered to men and women over 60, and its effects on muscle function and safety were assessed. 

## 2. Materials and Methods

### 2.1. Ethics

This study was a 12-week, randomized, double-blind, placebo-controlled performed at Jeonbuk National University Hospital, Jeonju-si, Korea. The study protocol and consent form were approved by the institutional review board (IRB No. 2022-01-075, accessed on 25 March 2022). It was registered in the Clinical Research Information Service (Cris), Republic of Korea (KCT0009883, https://cris.nih.go.kr/cris/search/detailSearch.do/28394, accessed on 25 March 2022).

### 2.2. Participants

Eligible participants included men and women aged 60 years and older. Comprehensive information on the study objectives, procedures, and potential risks was provided during an initial screening, and written informed consent was obtained from all participants. A total of 100 individuals were enrolled and randomized equally into treatment and placebo groups using SAS^®^ software (Version 9.4; SAS Institute, Cary, NC, USA) to generate a concealed randomization sequence. Baseline assessments, such as blood sampling, physical examinations, and medical history reviews, were conducted to ensure eligibility, with participants required to have a SARC-CalF score below 11 to commence the intervention within 14 days. 

### 2.3. HB05P and Placebo Capsules

The participants were assigned to receive either HB05P (containing 1 × 10^10^ cells/day) or placebo capsules (421.4 mg microcrystalline cellulose). The HB05P and placebo capsules weighed 430 mg and contained silicon dioxide and magnesium stearate (Table 1). The subjects were instructed to take one capsule daily with water throughout the 12-week duration. 

### 2.4. Analysis of Muscle-Related Factors

Muscle strength was measured using a Biodex System 3 Pro (Biodex, Shirley, NY, USA) at an angular velocity of 60°/s, with peak torque (TQ) and TQ relative to body weight (TQ/BW) for knee extensors and flexors recorded. Participants underwent three practice sessions to familiarize themselves with the equipment, followed by four recorded trials, and the average of these trials was used for analysis. Grip strength was assessed using a Jamar dynamometer (Patterson Medical, Green Bay, WI, USA), and muscle mass was evaluated using dual-energy X-ray absorptiometry (Prodigy Fuga, GE). Functional performance measures included gait speed and the timed up-and-go test as part of the short physical performance battery [28]. Blood samples were collected to quantify follistatin, myostatin, and high-sensitivity C-reactive protein (hs-CRP) using an enzyme-linked immunosorbent.

### 2.5. Safety Assessment

Safety monitoring was conducted at baseline (week 0) and study completion (week 12), documenting any adverse events and measuring vital signs, such as blood pressure and heart rate. Additional safety parameters included body weight, urinalysis, and comprehensive blood panels to assess liver and kidney function.

### 2.6. Statistical Analysis

Data were analyzed using SAS software (Version 9.4; SAS Institute, Cary, NC, USA). Efficacy analyses were based on the per-protocol (PP) population, while safety analyses encompassed all participants who received at least one dose post-randomization. Comparisons of baseline characteristics utilized chi-square tests, Fisher’s exact tests, or independent *t*-tests. Within-group differences were evaluated using paired *t*-tests, and analysis of covariance (ANCOVA) was applied to adjust for potential confounders, such as lifestyle factors [18,19,20,21,22]. Results are presented as means ± standard deviations, with a significance threshold of *p* < 0.05.

## 3. Results

### 3.1. Participants

**Figure 1:**
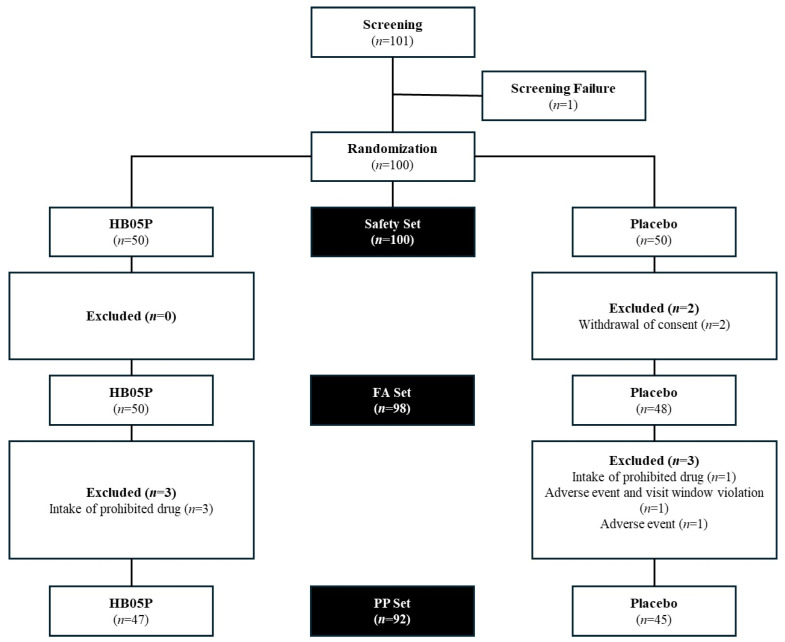
Flow diagram of the enrolled participants.

Of the 101 individuals initially enrolled, 100 were randomized equally into two groups, with 50 participants assigned to each (Figure 1). However, 2 participants withdrew from the study, resulting in 50 completing the trial in the HB05P group and 48 in the placebo group (FA set, *n* = 98). Due to protocol deviations, 6 participants from each group were excluded from the efficacy analysis, yielding a per-protocol population of 92 participants (47 in the HB05P group and 45 in the placebo group, PP set, *n* = 92).

### 3.2. Baseline Characteristics of Participants

Baseline demographic and lifestyle characteristics, including age, alcohol consumption, smoking status, and SARC-CalF scores, showed no significant differences between the groups (Table 2). Among the 100 participants, there were 12 men and 88 women (HB05P group: 5 men and 45 women; placebo group: 7 men and 43 women). The mean age of the cohort was 65.03 ± 3.83 years, with no notable disparities in age distribution. The average SARC-CalF score was 2.93 ± 4.35, consistent across both groups. 

### 3.3. Effect of HB05P on Isokinetic Muscular Strength 

**Figure 2:**
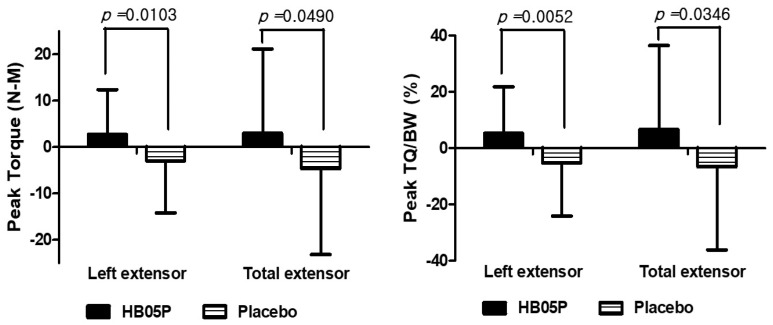
Change in extensor muscle strength. TQ/BW, peak torque/body weight.

Isokinetic muscle strength was assessed using isokinetic testing at baseline and after 12 weeks of intervention, with detailed results provided in Table 3. The analysis of lower limb strength changes, indicated by peak torque (maximum muscle strength), showed that participants in the HB05P group experienced significant gains in the strength of the left leg extensors and overall extensor muscles after 12 weeks. In contrast, the placebo group exhibited a reduction in these measures. The differences between the groups were statistically significant, with *p*-values of 0.0103 and 0.049, respectively (Figure 2). Moreover, the HB05P group demonstrated a significant increase in the TQ/BW ratio for the left leg extensors relative to baseline (*p* = 0.0354). The comparison of peak TQ/BW changes between the HB05P and placebo groups revealed significant differences, with *p*-values of 0.0052 for the left leg and 0.0346 for the combined legs (Figure 2).

### 3.4. Effect of HB05P on Grip Strength

Grip strength analysis did not reveal any significant differences between baseline and week 12 measurements in either group (Table 4).

### 3.5. Effect of HB05P on Follistatin and Myostatin Levels

**Figure 3:**
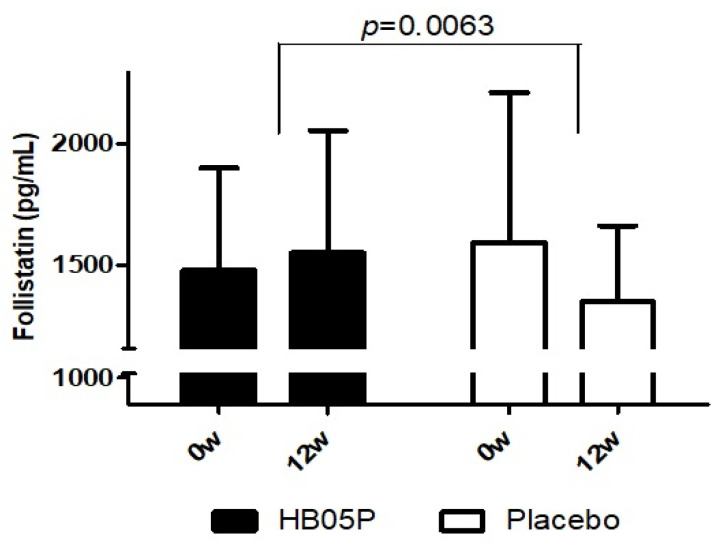
Comparison of change in blood follistatin levels following HB05P administration.

The secondary efficacy parameters, namely muscle-related hormones (follistatin and myostatin), were evaluated at baseline (prior to intake) and after 12 weeks of intervention during the third visit. The findings, shown in Table 5 and Figure 3, indicate a significant increase in serum follistatin levels in the HB05P group after 12 weeks compared to the baseline, while the placebo group showed a decrease from their baseline levels. A statistically significant difference was observed between the two groups (*p* = 0.0063). Conversely, no significant differences were observed in serum myostatin levels when comparing values at the baseline and after the 12-week period.

### 3.6. Assessment of the Safety of HB05P 

The incidence rates of adverse events are presented in Table 6. During the course of the study, 4 participants out of 100 who consumed the trial product reported mild or moderate adverse events: 1 case in the HB05P group and 3 cases in the placebo group, showing no statistically significant difference in the occurrence of adverse events between the groups (*p* > 0.05). The reported events included gum inflammation, COVID-19, chronic complex periodontitis, and flu-like symptoms. An evaluation of causality determined that none of these adverse events were associated with the trial product intake. Furthermore, an analysis of diagnostic medical test outcomes revealed no significant differences between the two groups (*p* > 0.05).

## 4. Discussion

We previously showed that pasteurized *Akkermansia muciniphila* significantly reduces muscle degradation markers, such as atrogin-1, in cultured C2C12 muscle cells [32]. In the present clinical study, we examined the effects of orally administered HB05P, a strain isolated from the breast milk of healthy Korean women, on muscle function in individuals aged 60 and older. This study was designed as a 12-week, randomized, double-blind, placebo-controlled trial, with participants receiving a daily dose of 10 billion HB05P cells, based on our prior experimental findings [32]. After 12 weeks, the treatment group exhibited a significant improvement in TQ and overall muscle strength, particularly in the left leg extensor muscles, compared to the placebo group. These outcomes suggest that *A. muciniphila* may be a valuable intervention for enhancing muscle performance in older adults.

Inflammatory cytokines reportedly activate the expression of atrogin-1 and MuRF1 ubiquitin ligases, key drivers of muscle protein degradation in skeletal muscle [33]. *A. muciniphila* reportedly stimulates the production of SCFAs, including acetate and propionate [25]. These SCFAs, produced by various gut microbiota, are associated with multiple health benefits, including potential improvements in muscle function. In germ-free (GF) mice models, which display reduced muscle mass and strength, administration of SCFAs or microbiota transplants can partially restore muscle structure and function [34]. Specifically, acetate is rapidly metabolized by muscle cells as an energy source [35]. 

Previous animal studies using a dexamethasone-induced muscle atrophy model in Sprague Dawley rats demonstrated a dose-dependent increase in grip strength, muscle mass, and muscle fiber area. The medium dose chosen for this human trial was determined based on safety guidelines established by the European Food Safety Authority [36].

Hormonal analysis revealed a significant elevation in follistatin levels in the treatment group compared to the placebo group (*p* = 0.0063). Follistatin is a key regulator of muscle regeneration, as it has been shown to promote the proliferation and differentiation of myoblasts, thereby enhancing tissue repair in animal models [37]. Recent studies have also identified follistatin as a binding partner of angiogenin, a critical factor in angiogenesis, indicating a possible role in vascular development and tissue repair [38]. Supporting this, the follistatin isoform 288 was reported to be essential for embryonic angiogenesis in mice [39]. The observed increase in follistatin is noteworthy, as it counteracts the inhibitory action of myostatin on muscle hypertrophy, thus contributing to enhanced muscle function.

## 5. Conclusions

The present study demonstrated that a 12-week administration of HB05P, in the absence of additional exercise, led to a significantly increased in muscle strength among individuals aged 60 years or older. Improved muscle function following HB05P administration is associated with increased follistatin levels. To the best of our knowledge, this is the first study showing that pasteurized *A. muciniphila* improves muscle function in humans, even without concomitant exercise. These results suggest potential therapeutic benefits of HB05P consumption for age-related muscle loss.

## Figures and Tables

**Table 1:** Description of the placebo and treatment products.

		Placebo	Treatment
Ingredients and content (%)	HB05P	-	11.63
	Microcrystalline cellulose	98.00	86.37
	Silicon dioxide	1.00	1.00
	Magnesium stearate	1.00	1.00
Packing	One capsule (430 mg) individually packed		

**Table 2:** Demographic and lifestyle characteristics of the participants at the baseline.

Variable	HB05P (*n* = 50)	Placebo (*n* = 50)	*p*-Value ^(1)^
Male (*n*, %)	5 (10.0)	7 (14.0)	0.5383 ^(2)^
Female (*n*, %)	45 (90.0)	43 (86.0)	
Age (year)	64.80 ± 4.25	65.26 ± 3.38	0.5507
Height (cm)	156.44 ± 6.71	157.68 ± 5.80	0.3256
Weight (kg)	59.87 ± 7.43	59.41 ± 7.80	0.7623
BMI (kg/m^2^)	24.44 ± 2.33	23.89 ± 2.86	0.2959
SBP (mmHg)	126.76 ± 12.84	129.82 ± 14.76	0.2714
DBP (mmHg)	74.70 ± 9.52	77.48 ± 11.15	0.1830
Pulse (bpm)	72.06 ± 7.57	70.20 ± 9.24	0.2735
SARC-Calf	2.84 ± 4.32	3.02 ± 4.43	0.8374
Alcohol (*n*, %)	8 (16.0)	10 (20.0)	0.6027 ^(2)^
Alcohol (unit, week)	2.23 ± 3.78	3.82 ± 6.24	0.5353
Current smoker (*n*, %)	0 (0.0)	1 (2.0)	>0.999 ^(3)^
Amount smoked (pieces/days)	-	12.00	

Values are presented as mean ± standard deviation; percentages may not total 100 due to rounding adjustments; *p*-values were analyzed using ^(1)^ an independent *t*-test, ^(2)^ chi-square tests, or ^(3)^ Fisher’s exact tests. BMI, body mass index; SBP, systolic blood pressure; DBP, diastolic blood pressure.

**Table 3:** Effects of HB05P on isokinetic muscular strength.

			Treatment Group (*n* = 47)			Placebo Group (*n* = 45)			*p*-Value
			Baseline	12 Weeks	*p*-Value	Baseline	12 Weeks	*p*-Value	
Peak Torque (N·m)	Left	Flexor	31.37 ± 17.12	31.33 ± 16.20	0.9869	34.68 ± 13.05	34.68 ± 15.92	0.9992	0.9892
		Extensor	68.72 ± 23.90	71.44 ± 21.70	0.0608	73.24 ± 21.05	70.24 ± 20.03	0.0794	0.0103
	Right	Flexor	32.62 ± 17.47	32.95 ± 16.62	0.8801	34.37 ± 16.97	36.08 ± 20.50	0.5009	0.6779
		Extensor	71.47 ± 23.88	71.74 ± 21.30	0.8750	75.70 ± 21.32	74.04 ± 24.69	0.4117	0.4627
Peak TQ/BW (%)	Left	Flexor	52.29 ± 28.05	52.60 ± 25.66	0.9426	59.02 ± 22.34	59.08 ± 27.03	0.9866	0.9642
		Extensor	115.06 ± 38.20	120.30 ± 35.85	0.0354	124.72 ± 35.56	119.40 ± 33.94	0.0635	0.0052
	Right	Flexor	54.15 ± 27.49	55.41 ± 27.01	0.7225	58.30 ± 28.92	60.84 ± 32.40	0.5433	0.8144
		Extensor	119.12 ± 36.19	120.51 ± 33.82	0.6078	128.79 ± 36.41	127.49 ± 36.00	0.5995	0.4634

TQ/BW, peak torque/body weight.

**Table 4:** Effects of HB05P on grip strength.

		Treatment Group (*n* = 47)			Control Group (*n* = 45)			*p*-Value ^(2)^
		Baseline	12 Weeks	*p*-Value ^(1)^	Baseline	12 Weeks	*p*-Value ^(1)^	
Hand grip (kg)	Left	23.50 ± 4.94	23.12 ± 5.46	0.3405	23.55 ± 6.41	23.96 ± 6.26	0.2644	0.1450
	Right	24.36 ± 5.03	23.94 ± 5.03	0.2651	24.33 ± 6.12	24.14 ± 5.78	0.6585	0.6861

Values are presented as mean ± SD. ^(1)^ Compared within groups; analyzed by paired *t*-test. ^(2)^ Compared between groups; analyzed by independent *t*-test.

**Table 5:** Effects of HB05P on hormones.

	Treatment Group (*n* = 47)			Control Group (*n* = 45)			*p*-Value ^(2)^
	Baseline	12 Weeks	*p*-Value ^(1)^	Baseline	12 Weeks	*p*-Value ^(1)^	
Follistatin (pg/mL)	1475.99 ± 424.63	1549.80 ± 506.44	0.3653	1591.14 ± 623.56	1345.89 ± 315.23	0.0039	0.0063
Myostatin (pg/mL)	13.37 ± 25.50	12.98 ± 26.50	0.5151	8.40 ± 19.86	9.03 ± 20.04	0.4430	0.3145

Values are presented as mean ± SD. ^(1)^ Compared within groups; analyzed by paired *t*-test. ^(2)^ Compared between groups; analyzed by independent *t*-test.

**Table 6:** Safety assessment at the baseline and after 12 weeks of HB05P and placebo intake.

	Treatment Group (*n* = 50)			Control Group (*n* = 50)			*p*-Value ^(2)^
	Baseline	12 Weeks	*p*-Value ^(1)^	Baseline	12 Weeks	*p*-Value ^(1)^	
WBC	5.23 ± 1.10	5.14 ± 1.31	0.5406	4.94 ± 1.32	4.99 ± 1.58	0.7270	0.4937
RBC	4.37 ± 0.32	4.39 ± 0.28	0.4764	4.32 ± 0.37	4.29 ± 0.37	0.1961	0.1456
Hemoglobin	13.39 ± 1.01	13.54 ± 0.88	0.0231	13.27 ± 0.93	13.26 ± 0.99	0.8460	0.0890
Hematocrit	41.45 ± 2.78	41.00 ± 2.32	0.0638	41.08 ± 2.76	40.39 ± 3.03	0.0282	0.5241
Platelet	240.68 ± 49.72	239.96 ± 49.14	0.8283	239.58 ± 44.26	244.80 ± 54.46	0.1567	0.2289
ALP	73.18 ± 15.90	75.94 ± 16.44	0.0124	76.82 ± 21.90	75.42 ± 21.30	0.2806	0.0142
Gamma-GTP	19.14 ± 9.10	18.06 ± 7.91	0.0197	21.28 ± 11.00	21.14 ± 11.55	0.8865	0.3845
AST	22.58 ± 6.65	22.36 ± 5.19	0.7636	22.94 ± 6.01	22.90 ± 6.40	0.9579	0.8640
ALT	18.88 ± 9.03	18.34 ± 7.21	0.5598	19.68 ± 8.69	19.86 ± 9.72	0.8684	0.6130
Total bilirubin	0.61 ± 0.23	0.65 ± 0.28	0.2051	0.64 ± 0.23	0.59 ± 0.17	0.0271	0.0247
Total protein	7.20 ± 0.32	7.24 ± 0.34	0.2738	7.21 ± 0.36	7.16 ± 0.33	0.1966	0.0898
Albumin	4.56 ± 0.22	4.58 ± 0.21	0.5741	4.58 ± 0.21	4.58 ± 0.20	0.7275	0.5150
BUN	15.76 ± 4.25	14.66 ± 4.32	0.0216	15.00 ± 4.43	14.96 ± 4.23	0.9423	0.1436
Creatinine	0.68 ± 0.15	0.68 ± 0.14	0.8872	0.67 ± 0.11	0.67 ± 0.12	0.6591	0.6742
Glucose	87.10 ± 7.72	86.56 ± 6.98	0.6348	89.86 ± 11.23	90.88 ± 9.43	0.3639	0.3277
CK	107.38 ± 51.68	100.56 ± 42.89	0.3693	94.12 ± 39.38	100.38 ± 56.39	0.4130	0.2237
LD	205.28 ± 28.37	204.12 ± 25.98	0.6999	196.04 ± 23.84	202.54 ± 24.49	0.0125	0.0525

Values are presented as mean ± SD. ^(1)^ Compared within groups; analyzed by paired *t*-test. ^(2)^ Compared between groups; analyzed by independent *t*-test. WBC, white blood cell; RBC, red blood cell; ALP, alkaline phosphatase; GTP, glutamyl transferase; AST, aspartate transaminase; ALT, alanine transaminase; BUN, blood urea nitrogen; CK, creatine kinase; LD, lactate dehydrogenase.

## Data Availability

The datasets generated during and/or analyzed during the current study are available from the corresponding author on reasonable request due to ethical.
